# Accumulation and Release of Cadmium Ions in the Lichen *Evernia prunastri* (L.) Ach. and Wood-Derived Biochar: Implication for the Use of Biochar for Environmental Biomonitoring

**DOI:** 10.3390/toxics12010066

**Published:** 2024-01-13

**Authors:** Andrea Vannini, Luca Pagano, Marco Bartoli, Riccardo Fedeli, Alessio Malcevschi, Michele Sidoli, Giacomo Magnani, Daniele Pontiroli, Mauro Riccò, Marta Marmiroli, Alessandro Petraglia, Stefano Loppi

**Affiliations:** 1 Department of Chemistry, Life Sciences, and Environmental Sustainability, University of Parma, Parco Area delle Scienze 11/a, 43124 Parma, Italy; luca.pagano@unipr.it (L.P.); marco.bartoli@unipr.it (M.B.); alessio.malcevschi@unipr.it (A.M.); marta.marmiroli@unipr.it (M.M.); alessandro.petraglia@unipr.it (A.P.); 2 National Interuniveritary Consortium for Environmental (CINSA), University of Parma, Parco Area delle Scienze 95, 43124 Parma, Italy; 3 Department of Life Sciences, University of Siena, Via PA Mattioli 4, 53100 Siena, Italy; riccardo.fedeli@student.unisi.it (R.F.); stefano.loppi@unisi.it (S.L.); 4 Department of Mathematical, Physical and Computer Sciences, University of Parma, Parco Area delle Scienze 7/a, 43124 Parma, Italy; michele.sidoli@unipr.it (M.S.); giacomo.magnani@unipr.it (G.M.); daniele.pontiroli@unipr.it (D.P.); mauro.ricco@unipr.it (M.R.); 5 BAT Center-Interuniversity Center for Studies on Bioinspired Agro-Environmental Technology, University of Naples ‘Federico II’, 80138 Napoli, Italy

**Keywords:** biomonitoring, cadmium accumulation, cadmium release, cadmium removal, cation exchange capacity, surface area

## Abstract

Biochar (BC) boasts diverse environmental applications. However, its potential for environmental biomonitoring has, surprisingly, remained largely unexplored. This study presents a preliminary analysis of BC’s potential as a biomonitor for the environmental availability of ionic Cd, utilizing the lichen *Evernia prunastri* (L.) Ach. as a reference organism. For this purpose, the lichen *E. prunastri* and two types of wood-derived biochar, biochar 1 (BC1) and biochar 2 (BC2), obtained from two anonymous producers, were investigated for their ability to accumulate, or sequester and subsequently release, Cd when exposed to Cd-depleted conditions. Samples of lichen and biochar (fractions between 2 and 4 mm) were soaked for 1 h in a solution containing deionized water (control), 10 µM, and 100 µM Cd^2+^ (accumulation phase). Then, 50% of the treated samples were soaked for 24 h in deionized water (depuration phase). The lichen showed a very good ability to adsorb ionic Cd, higher than the two biochar samples (more than 46.5%), and a weak ability to release the metal (ca. 6%). As compared to the lichen, BC2 showed a lower capacity for Cd accumulation (−48%) and release (ca. 3%). BC1, on the other hand, showed a slightly higher Cd accumulation capacity than BC2 (+3.6%), but a release capacity similar to that of the lichen (ca. 5%). The surface area and the cation exchange capacity of the organism and the tested materials seem to play a key role in their ability to accumulate and sequester Cd, respectively. This study suggests the potential use of BC as a (bio)monitor for the presence of PTEs in atmospheric depositions and, perhaps, water bodies.

## 1. Introduction

Cadmium (Cd) is a relatively abundant metal in the Earth’s crust (about 0.1 mg/kg; [1]). However, its continuous release from anthropogenic activities [2] can increase its concentration in soils, atmospheric depositions, and water bodies [3]. Cadmium is a non-essential metal for human health. However, its chronic presence at concentrations above environmental background levels can cause several adverse human health effects, including, in the worst case, the development of various types of cancer [4,5,6]. Due to its high chemical and physical similarity to calcium (Ca; [7], Cd is easily translocated in the human body, where it can remain for decades (t_1/2_ = 10–35 years; [8]. For this reason, Cd is recognized as a global priority pollutant. Daily consumption of contaminated vegetables is the main route by which the human body is exposed to the metal [9]. Therefore, its threshold concentration in vegetables is set at 0.1 mg/kg fresh weight, at the European level [10]. Cases of poisoning and serious illness resulting from the consumption of Cd-contaminated plant material due to Cd contamination of air and water (e.g., Itai-Itai disease) are well known. Cadmium emissions must therefore be controlled, and, in this scenario, Europe has set limits on particulate emissions from industrial activities [11], which has led to a decrease in Cd emissions [12]. The reduction in emissions has also helped to reduce the atmospheric deposition of Cd [13], although some countries, such as Poland, Slovakia, and Hungary, still have high levels [14,15].

Epiphytic lichens are an effective way to assess the presence of potentially toxic elements (PTEs) in atmospheric depositions [16,17,18,19]. Due to the lack of roots, protective structures such as plant cuticles, and gas flow regulating structures such as stomata, lichens can efficiently accumulate airborne PTEs far beyond their physiological needs and without regard to their phytotoxicity [20,21,22]. Accumulation occurs when PTE levels in atmospheric deposition exceed their natural background concentrations but ends when equilibrium with their new environmental availability is reached [23]. On the other hand, when the environmental availability of PTEs decreases, as can occur after environmental improvements [24], PTE releases become the dominant process and thalli experience a decrease in their concentrations [23]. Lichens require long periods of time to release the accumulated metal [22,24], but in some cases, i.e., for some specific chemical species of metals such as mercury, the release appears to be irreversible [25]. There is extensive research in the literature on the ability of lichens to accumulate Cd (see [20,26] for examples), but there is no information on the fate of this metal after its environmental availability has been reduced.

Biochar (BC), the by-product of the pyrolysis of organic materials (often wood) for bioenergy production, is now attracting considerable attention, from an environmental perspective, as a strategy to stabilize PTEs in soils [27,28,29], thereby enabling their remediation [30]; as a tool for filtering contaminated water and air [31,32,33]; and as a sustainable strategy for agricultural purposes [34,35]. Despite these diverse applications, the potential of BC for environmental biomonitoring has been surprisingly underexplored.

To assess the suitability of BC for biomonitoring, akin to assessments conducted with lichens, it is essential to understand its capacity to reflect environmental concentrations of PTEs, including Cd. While BC’s documented ability to accumulate Cd through various mechanisms is extensive [36], its desorption capacity remains less explored [37]. This study presents a preliminary analysis of BC’s potential as a biomonitor for the environmental availability of ionic Cd, utilizing the lichen *Evernia prunastri* as a reference organism. To this end, the dynamics of the accumulation and release of Cd^2+^ in the lichen *E. prunastri* (a well-known biomonitor of environmental PTE availability) and in two types of biochar were compared through an experiment conducted in a controlled environment.

## 2. Materials and Methods

### 2.1. BC Samples Description and Pretreatment

Samples of both biochar 1 (BC1) and biochar 2 (BC2) BC were provided by the respective (anonymous) producers. Both materials were produced from woody biomass of forestry origin pyrolyzed at 500–650 °C. BC1 was produced using a 125 kWe industrial gasifier while BC2 was produced using a Cryos Gas Unit plant. BC1 corresponded to material produced and disposed (i.e., waiting for disposal) in non-waterproof plastic bales exposed to the elements (i.e., rain, sun, and temperature variation) for 5 years.

**Figure 1:**
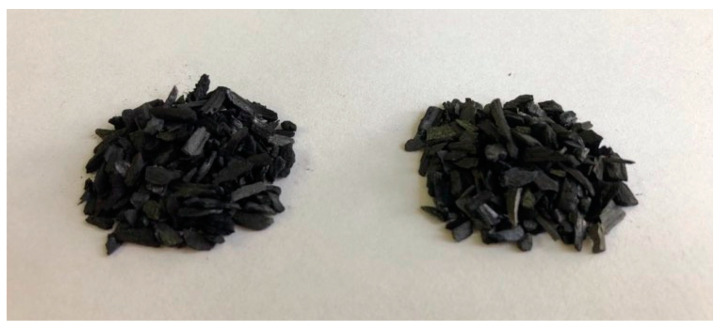
Sieved (2–4 mm) samples of BC1 (**left**); and BC2 (**right**).

BC1 and BC2 samples were first sieved at 4 mm and then at 2 mm to obtain materials with a flake size between 2 and 4 mm (Figure 1). The samples were then separately subjected to a preliminary wash with deionized water (1.1 µS/cm^2^) to remove the first soluble component adsorbed on the BC, such as salts, followed by a wash with warm 1M HCl maintaining a sample mass (g)/solution volume (mL) ratio of 1:250; this procedure was considered to remove most of the metals, including Cd, from the BC surface. The BC samples were then air-dried at 80 °C overnight and stored in polypropylene tubes. The pool of BC1 and BC2 material (60 g each) was divided into 30 samples, each weighing 2 g. Each sample was subsequently enclosed in a nylon mesh with a 0.2 mm mesh size and closed at one end using nylon thread.

### 2.2. Lichen Sample Collection and Preparation

Thalli of the lichen *Evernia prunastri* (L.) Ach. were collected from branches of *Quercus* spp. located in a remote forest area in southwestern Emilia-Romagna (October 2022). For this study, only adult thalli (laciniae > 5 cm) growing on branches more than 1.5 m above the ground were collected. In the laboratory, samples were cleaned of any foreign material, such as bark debris, spider nests, and other lichen species.

Lichen samples were slowly hydrated overnight in a climatic chamber at 90% and then washed with deionized running water (1.1 µS/cm^2^) to remove adhering dust and to hypothetically create a pool of lichen material with similar elemental concentrations [18]. The lichen material (60 g) was divided into 30 samples, each weighing 2 g and then underwent the same procedure as those of the BC.

### 2.3. Surface Area of Lichen and Biochar Samples

The specific surface area (SSA) measurement was conducted on both BCs and *E. prunastri* using the methylene blue (MB) adsorption method. This measurement involves determining the SSA of the materials under investigation by quantifying the amount of MB molecules adsorbed on the surface of the analyte through UV-Vis spectroscopy in a water environment [38,39,40]. After proper calibration to establish the correlation between absorbance and dye concentration, the analysis was performed by mixing 10 mg of the analyte (samples not treated with Cd) with 10 mL of MB solutions at various concentrations (ranging from 1 × 10^−6^ M to 3 × 10^−4^ M) and stirring for 24 h. Subsequently, the suspensions were centrifuged for 30 min at 5000 rpm to separate the solid fraction. The supernatant solutions were then measured using a Jasco V-550 UV–Visible spectrophotometer to assess the differences in MB concentration after coming into contact with the analytes, thereby determining the SSA of the samples under investigation (refer to the Supporting Information for more details).

### 2.4. Cation Exchange Capacity Measurement

Samples not treated with Cd (2 g) were transferred to 50 mL centrifuge tubes with 30 mL of a pH 8.2 buffered barium chloride (BaCl_2_) solution. Subsequently, the tubes were centrifuged at 3000× *g* rpm for 1 h. The resulting supernatant was decanted into a 100 mL tared flask. This process was iterated twice, with the clear supernatants being collected in the same 100 mL flask. The final volume was adjusted using the pH 8.2 buffered BaCl_2_ solution. A comprehensive sample wash was conducted by adding 30 mL of water, followed by centrifugation. Using a precision burette, 30 mL of a 50 mM magnesium sulfate (MgSO_4_) solution was carefully introduced into the centrifuge tubes. The tubes were subsequently manually agitated to ensure complete dispersion of the sample. Continuous agitation was maintained for 2 h, followed by centrifugation. Ten milliliters of the resulting clear solution were extracted and transferred into a 250 mL flask. To this, 100 mL of distilled water, 10 mL of an ammonium chloride (NH_4_Cl) buffer solution, and a minimal amount of indicator were added to create the sample solution. Concurrently, a blank test solution was prepared by transferring 100 mL of distilled water, 10 mL of the 50 mM MgSO_4_ solution, 10 mL of an NH_4_Cl buffer solution, and a small quantity of indicator into a separate 250 mL flask. Both the blank test solution and the sample solution were titrated using a 2.5 cmol/L ethylendiaminotetracetyc acid (EDTA) solution until the appearance of a distinct blue color, signifying the titration endpoint. Results were expressed as meq/100 g. See the Gazzetta ufficiale della Repubblica Italiana, Serie Generale No. 248 of 21 October 1999 of the Ministero delle Politiche Agricole e Forestali for full details [41].

### 2.5. Treatments with Cd^2+^ and Depuration Phase

Samples were separately immersed for 1 h in solutions containing Cd^2+^ (CdCl_2_) at concentrations of 0 (control), 10, and 100 µM (accumulation phase), maintaining a sample mass (g)/solution volume (mL) ratio of 1:250. In one hour, both BCs and lichens show (approximately) their maximum Cd^2+^ accumulation capacity [42,43]. After their exposure, all samples were blotted with paper towels to remove the excess water and then left overnight in ambient conditions. Half of each set of the treated samples were then separately soaked in deionized water for 24 h (depuration phase) maintaining a sample mass (g)/solution volume (mL) ratio of 1:250. The samples were blotted with paper towels and dried overnight at 40 °C. All the samples were then stored in vacuum bags at −15 °C until the analysis.

### 2.6. Sample Analysis

Sample analyses were performed according to [44], with modifications. Lichen samples (2 g) were solubilized with 9 mL HNO_3_ (65%) and 1 mL of H_2_O_2_ at 165 °C for 20 min using a heated digester thermoblock (DK20, Velp Scientifica, Usmate Velate, MB, Italy). Specifically, samples were first solubilized for 10 min with 9 mL of HNO_3_ and then with the addition of 1 mL of H_2_O_2_ for a further 10 min. The solubilized samples were collected in 15 mL tubes and left at room temperature until the analysis. BC samples (2 g) were first incinerated at 450 °C for 24 h and then solubilized as described above; samples were considered ready for acid solubilization when they no longer showed a black discoloration. All digested samples were filtered at 0.22 μm. Cadmium concentrations were quantified by flame atomic absorption spectrometry (AA240FS, Agilent Technologies, Santa Clara, CA, USA). The Cd calibration curve was prepared by diluting 1000 ppm certified standard solutions (Agilent Technologies, Santa Clara, CA, USA). Cadmium concentrations were expressed as mg/kg. Three technical replicates were performed for each sample. Samples with concentrations below the limit of quantification (0.05 mg/kg), such as Cd-untreated samples, were analyzed by ICP-MS (Perkin Elmer-Sciex, Elan 6100; Cd detection limit <0.001 mg/kg). The analytical quality was evaluated using the certified reference material NCS DC73350 “Leaves of Poplar”. An average accuracy higher than 95% and a precision of 98% were obtained.

Losses of Cd^2+^ resulting from the incineration step were assessed by comparing the Cd content of BC samples analyzed after the 450 °C process with their direct (albeit partial) chemical digestion using HNO_3_ and H_2_O_2_ (9/1 *v*/*v*); see Section 2.6 for details. The recovery of Cd was always higher than 94%.

### 2.7. Statistics

Due to the low number of statistical replications (*n* = 6), the search for statistically significant differences between the treatments was carried out by means of permutation tests for multiple comparisons, using the Benjamini–Hochberg correction. All calculations were performed through R software [45].

## 3. Results and Discussion

**Figure 2:**
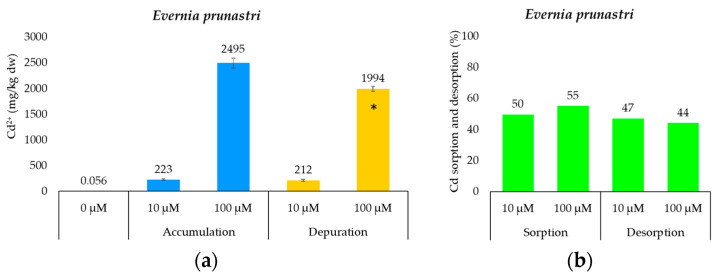
(**a)** concentration of Cd (mean ± standard deviation) in samples of the lichen species *Evernia prunastri* exposed to 10 and 100 μM Cd^2+^ solutions (accumulation phase) and then subjected to a depuration phase (only deionized water) for 24 h; the values above the bars are the averages of the six replicates analyzed. Legend: * indicates statistically significant differences in Cd concentration (for each exposure concentration) between the ‘accumulation’ and ‘depuration’ phases (*p* < 0.05; N = 6); and (**b**) cadmium sorption and desorption (%) in lichen samples first exposed to Cd and then exposed to the purification step, respectively. The values above the bars represent the percentages of Cd sequestered and therefore present on the surface of lichen after the accumulation and purification treatments.

The concentration of Cd measured in samples of *E. prunastri* before (background concentrations) and after their exposure to two different concentrations of Cd (accumulation phase) as well as after their immersion in a Cd-free solution (depuration phase) are reported in Figure 2.

Unexposed lichen samples (control samples) showed Cd concentrations consistent with those commonly found in lichens from remote areas (i.e., 0.14 ± 0.03 mg/kg_dw_ from Cecconi et al. [46] and 0.21 ± 0.17 mg/kg_dw_ from Vannini et al. [47]). On the other hand, Cd-exposed samples showed concentrations several orders of magnitude higher than those commonly found in unpolluted thalli, thus confirming the efficiency of lichens to accumulate ionic Cd in proportion to its simulated environmental availability. Specifically, the concentrations of Cd measured within the lichen *E. prunastri* treated with 10 uM Cd solutions are approximately 32 times higher than those detectable in thalli of the fruticose lichen *Ramalina farinacea* exposed to emissions from a lignite power plant [48] and many orders of magnitude higher than those measured in thalli of *Parmelia sulcata* native to a mining area in Ghana [49]. Nevertheless, Cd concentrations measured in treated thalli of *E. prunastri* are much higher than those measured in thalli of the same lichen species treated with the same concentrations of Cd used in this experiment, albeit after a shorter exposure time (about 30 min; [50]). In detail, Sujetovienė and Šliumpaitė [50] measured Cd concentrations ca. 2.5 times lower than those measured in this study after the treatment with 10 μM Cd^2+^. These differences are probably due to the different treatment the samples underwent before total chemical analysis, which in the case of Sujetovienė and Šliumpaitė [50] was a rapid washing of the samples with deionized water immediately after the accumulation phase. This step, which was not carried out in this study, likely hindered lichen samples from accumulating the metal more efficiently. This is because the part of the metal not yet bound to the active sites of the organism would have been immediately removed by washing. After all, the lichen exhibits an excellent ability to accumulate the metal from the exposure solution, and this capacity seems to be independent of the provided metal concentration. In fact, a calculation aimed at estimating the percentage of metal sequestered by the lichen following its exposure to the lowest (10 μM) and highest (100 μM) treatment concentrations indicates a metal sequestration capacity of 50% and 55%, respectively. These results are quite consistent with those reported by Paoli et al. [26] for the lichen *Xanthoria parietina*.

The accumulation of ionic forms of elements within lichens is the result of both intracellular and extracellular processes [51]. Intracellular accumulation occurs when an element has lower affinity for extracellular exchange groups (i.e., carboxylic and hydrocarboxylic groups and chitin; [52]) compared to intracellular ones. On the other hand, extracellular accumulation occurs through the chelating action of oxalates produced by the mycobiont, along with ionic bonding with the cell wall of the fungal and algal partners [53]. When compared to other essential elements for metabolism such as Cu, for example, Cd exhibits higher rates of extracellular accumulation than intracellular rates [26,54].

The accumulation of metals within lichens is, however, a partially reversible process [55]; in fact, compared to lichen samples exposed to the accumulation phase, samples exposed to the purification phase (24 h) showed a non-significant (about 3%; *p* > 0.05) as well as a significant Cd release (i.e., about 9%) when exposed to the lowest and highest Cd concentrations, respectively, with an average Cd release of about 6%. The absence of a significant release of ionic Cd^2+^ from samples exposed to the lowest treatment concentration may indicate an excellent ability of the lichen to accumulate the metal irreversibly, at least over the 24 h period tested; this result could be attributed to the incomplete saturation of the lichen’s binding sites for Cd. Instead, the statistically significant reduction in the Cd concentration from thalli treated with the highest treatment concentration (100 μM) may be due to the release of Cd that was not effectively immobilized in the thallus. In addition, however, the reduction in Cd content after exposure to the purification phase could also be due to partial release of the metal temporarily accumulated intracellularly due to an increase in its extracellular concentration, a process observed in both *Peltigera rufescens* and *Cladina arbuscula* [20]. In fact, Loppi et al. [56] clearly reported that the reduction in PTE content 24 h after the purification phase was mainly due to the release of the metal from the intracellular level of the lichen, while its content at the extracellular level remained unchanged. Cadmium exposure easily activates defense responses at the photobiont level (such as phytochelatin activation and protein synthesis; [57]) to avoid physiological impairment [58], mechanisms that are probably involved in the dynamics assumed above. Cadmium is, in fact, a non-essential element for the metabolism of lichens (or living organisms in general), which easily justifies the low rate of intracellular uptake (μmol/g/h) in lichen thalli as compared to their extracellular uptake [42].

**Figure 3:**
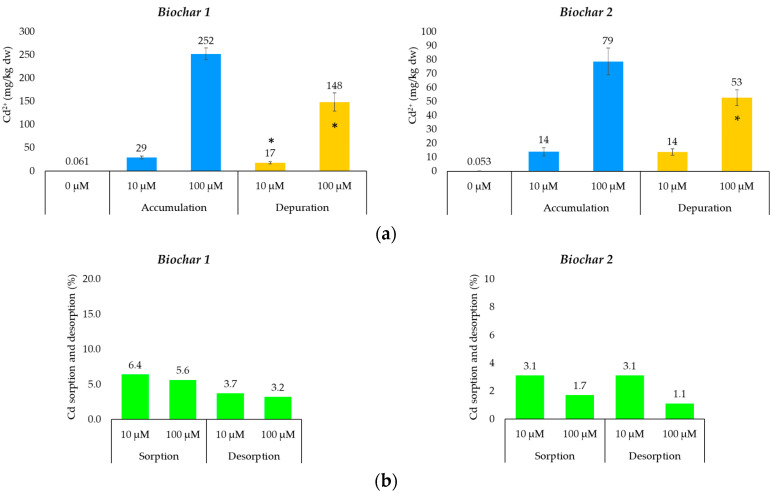
(**a**) concentration of Cd (mean ± standard deviation) in sample of the aged (BC1) and new (BC2) wood-derived biochar exposed to 10 and 100 μM Cd^2+^ solutions (accumulation phase) and then subjected to depuration phase (only deionized water) for 24 h; the values above the bars are the averages of the six replicates analyzed. Legend: * indicates statistically significant differences in Cd concentration (for each exposure concentration) between the ‘accumulation’ and ‘depuration’ phases (*p* < 0.05; N = 6); and (**b**) cadmium sorption and desorption (%) in in BC1 and BC2 samples first exposed to Cd and then exposed to the purification step, respectively. The values above the bars represent the percentages of Cd sequestered and therefore present on the surface of the BC after the accumulation and purification treatments.

Figure 3 shows the concentration of Cd measured in BC1 and BC2 before (background concentrations) and after their exposure to two different concentrations of Cd (accumulation phase) as well as after their immersion in a Cd-free solution (depuration phase).

Both unexposed biochar samples showed Cd concentrations consistent with each other and with unexposed lichen samples. In particular, the Cd concentration in both unexposed BC samples was below the maximum acceptable level for organic certification (0.7 mg/kg; [59]), the most stringent level for the use of BC in agriculture. After exposure to the accumulation phase, BC1 showed Cd accumulation proportional to the treatment concentration, whereas BC2 did not. In fact, the Cd concentration measured in BC2 samples after the treatment with the highest Cd concentration was only six times higher than that measured after the treatment with the 10 µM Cd^2+^ solution. The Cd content measured in BC samples treated with 10 μM Cd^2+^ solution aligns with the adsorption capacity of the material for the metal studied (0.3–39.1 mg/kg; [60]), thus confirming the ability of BC as a sorbent material for the removal of PTEs from water bodies. On the other hand, BCs exposed to the highest Cd concentration (100 μM; i.e., not environmentally relevant) easily exceeded this range.

After being subjected to the purification step, BC1 and BC2 showed a different ability to retain the accumulated metal. Specifically, BC1 showed a constant (ca. 40%) capacity to release Cd, regardless of its initial ionic concentration, whereas BC2 released the metal significantly (*p* < 0.01) only after treatment with 100 μM Cd; no Cd release was measured in BC2 samples treated with 10 μM Cd^2+^ and exposed to the depuration phase (*p* > 0.05). These results suggest that BC2 seems to be able to accumulate the metal stably (but not permanently), at least when adsorbed up to a concentration of about 14 mg/kg. Since BC1 showed a Cd concentration of 17 mg/kg after the purification step, this value could perhaps be considered as the Cd concentration that biochar (in a general sense) would be able to sequester stably after 24 h, provided there is no competition for exchange sites with other elements [26]. The presence of other metals in the solution could reduce the accumulation of Cd on the surface of BC, thereby diminishing its ability to sequester the metal [61].

Looking at Figure 2, both BCs showed a different ability to accumulate and release Cd, with the former (BC1) appearing to accumulate it more efficiently than the latter (BC2), although it has little capacity to retain it once adsorbed. Specifically, BC1 showed an ability to accumulate Cd in an ionic form that was approximately three times greater (*p* < 0.001; Student’s *t*-test) than that of BC2 when exposed to high Cd concentrations (100 μM) and approximately two times greater (*p* < 0.001; Student’s *t*-test) when exposed to environmentally relevant concentrations (10 μM). However, when the concentrations compared were those measured after the depuration phase, BC2 showed a better ability to retain Cd than BC1 only when exposed to very high concentrations of the metal. Therefore, BC1, having both a greater capacity to accumulate Cd and to release it once its environmental concentration decreases compared to BC2, could be tested in the future as a biomonitor, for the environmental availability of heavy metals such as Cd, dissolved in aquatic environments. Comparing the results of Cd accumulation in BC1 with those obtained from *E. prunastri* lichens, the latter have a greater capacity to both accumulate and retain the metal in ionic form (*p* < 0.001; Student’s *t*-test).

Table 1 summarizes the results of the surface area analysis (m^2^/g) calculated for each of the materials studied before their exposure to Cd, see the corresponding curves (Figures S1–S3) in the Supplementary Materials File.

**Figure 4:**
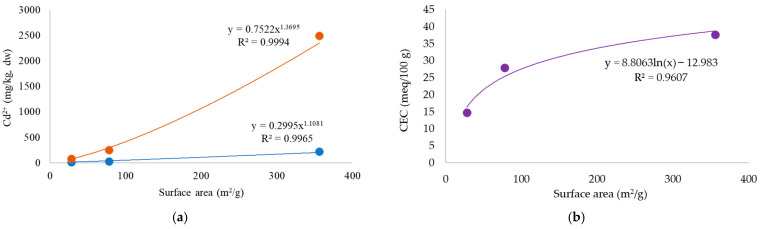
(**a**) correlation between surface area (m^2^/g) and Cd content (mg/kg) for the environmental matrices tested exposed to the lower (10 µM; blue line and dots) and higher (100 µM; brown line and dots) Cd concentration; and (**b**) correlation between surface area (m^2^/g) and CEC (meq/100 g) for the environmental matrices tested before their exposure to Cd.

It is clear from the table that the three materials studied in terms of surface area follow the hierarchy: lichen > BC1 > BC2, results that are clearly consistent with the hierarchy described above for Cd accumulation capacity: i.e., lichen > BC1 > BC2. Therefore, the parameters Cd accumulation and surface area appear to be positively correlated, albeit in a power rather than a linear relationship (Figure 4).

The greater capacity of BC1 than BC2 to accumulate Cd could be due to its ‘seniority’. Ageing increases the water-holding capacity of BC [62] and therefore its ability to quantitatively sequester elements such as Cd [63]. Consistent with this, BC1 has a lower density than BC2, confirming the higher surface area measured compared to that of BC2. Biochemical ageing can also increase the amount of oxygenated functional groups on the BC surface, although without increasing the surface area [64], thus providing an additional sequestration surface for Cd. Indeed, the importance of these groups for the removal of Cd from contaminated sites is well known [65,66]. However, it is important to specify that, since these two types of BC came from different biomass sources, the differences in Cd accumulation between the two investigated BCs may simply be due to variations in their chemical and physical characteristics [67,68], such as surface area [69] and cation exchange capacity (CEC, i.e., the substrate’s ability to exchange ions), parameters that are closely related. This relationship was observed among the three materials analyzed, although it follows a logarithmic trend rather than a linear one. This observation confirms what was previously noted: as the surface area increases, so does the material’s capacity to adsorb (and subsequently exchange) PTEs such as Cd. It is reasonable to speculate that the activation of BC, in addition to increasing the sample’s surface area [70], may also enhance its CEC [71], thereby improving its ability to adsorb PTEs. Comparing the PTE-accumulation capacity between lichen and activated BC would certainly yield intriguing results, given that activation can significantly increase BC’s surface area. As a result, all parameters related to Cd sequestration, such as complexation, physical absorption, cation exchange capacity, precipitation, and electrostatic absorption [60], would be amplified. Future indications regarding the differential capacity for the accumulation of PTEs in atmospheric depositions between the lichen *E. prunastri* and activated BC could shed light on the potential use of BC to assess the environmental availability of PTEs in atmospheric depositions primarily associated with particulate matter (PM) [18]. These findings could be of considerable interest in testing other valid surrogates for this purpose [72]. Indeed, as far as we know, research on BC’s ability to trap PM from atmospheric depositions seems to be an underexplored topic.

Currently, the use of biochar in the environmental field concerns both the reduction of PTE mobility in soils to increase the edibility of crop products [28,29] and the removal of heavy metals from wastewater [31,32]. However, based on the results obtained in this study, its ability to provide information on the environmental availability of heavy metals such as Cd should not be ruled out, especially when it comes to water bodies. Its use for soil monitoring would, therefore, be unnecessary, as this matrix can be easily sampled using traditional methodologies. By exposing biochar to a water body for a defined period (i.e., one month), it would be possible (this is purely speculative for now) to obtain integrated information on the availability of the chemical elements in the water body under investigation, without having to resort to numerous point source analyses of the water body, which may provide an accurate indication of element concentrations, but require numerous monitoring campaigns. Using this approach, biochar could be used in a very similar way to lichens, i.e., to provide information on the environmental availability of PTEs during the exposure period, but in the aquatic rather than the atmospheric compartment. Specific work would therefore be useful to clarify the feasibility of using this substrate to monitor the integrated availability of PTEs in streams of environmental interest.

## 4. Conclusions

Biochar has various environmental applications. However, its largely unexplored potential for environmental biomonitoring has prompted us to fill this gap. This study provides an initial analysis of how BC could serve as a biomonitor for the environmental availability of ionic Cd, using the lichen *Evernia prunastri* (L.) Ach. as a reference organism. The lichen showed a very good ability to adsorb ionic Cd—higher than the two biochar samples (more than 46.5%)—and a weak ability to release the metal (ca. 6%). As compared to the lichen, BC2 had a lower capacity for Cd accumulation (−48%) and release (ca. 3%). BC1, on the other hand, showed a slightly higher Cd accumulation capacity than BC2 (+3.6%), but a release capacity similar to that of the lichen (ca. 5%). Specific information regarding the dynamics of PTE accumulation and release in BC requires further investigation. The surface area and the CEC of the organisms/materials being tested seem to play a key role in their ability to accumulate Cd. Based on the results obtained, this work suggests the potential use of biochar as a (bio)monitor for the presence of PTEs in atmospheric depositions as well as in water bodies, when activated by, for example, increasing their surface area to enhance the material’s capacity to accumulate the target metal.

## Figures and Tables

**Table 1:** Estimated surface area (m^2^/g) and relative standard error as well as, cation exchange capacity (CEC), and density, of the lichen *Evernia prunastri*, biochar 1 (BC1), and biochar 2 (BC2).

Samples	Surface Area (m^2^/g) ± Std. Dev.	Cation Exchange Capacity (CEC; meq/100 g)	Density (g/cm^3^)
Lichen (*E. prunastri*)	356 ± 26	37.7	-
Biochar 1 (BC1)	78 ± 10	28.0	0.21
Biochar 2 (BC2)	28 ± 2	14.8	0.44

## Data Availability

The raw data presented in this study are available on request from the corresponding author.

## Supplementary Materials

The following supporting information can be downloaded at: https://www.mdpi.com/article/10.3390/toxics12010066/s1, Figure S1: Langmuir isotherm fit for BC1 (new) sample; Figure S2: Langmuir isotherm fit for BC2 (aged) sample; Figure S3: Langmuir isotherm fit for *Evernia prunastri* (L.) Ach. sample.
