# Supplementary material for: Accumulation and Release of Cadmium Ions in the Lichen Evernia prunastri (L.) Ach. and Wood-Derived Biochar: Implication for the Use of Biochar for Environmental Biomonitoring
Source: Toxics. 2024 Jan 13;12(1):66. doi: 10.3390/toxics12010066 (PMC10818847; doi:10.3390/toxics12010066)
Supplement: Supplementary file 1 [file toxics-12-00066-s001.zip › toxics-2794081-supplementary.pdf]

Accumulation and Release of Cadmium Ions in the Lichen *Evernia prunastri* L. (Ach.) and Wood-Derived Biochar: Implication for the Use of Biochar for Environmental Biomonitoring

Andrea Vannini [1,*], Luca Pagano [1,2], Marco Bartoli [1], Riccardo Fedeli [3], Alessio Malcevschi [1], Michele Sidoli [4], Giacomo Magnani [4], Daniele Pontiroli [4], Mauro Riccò [4], Marta Marmiroli [1,†], Alessandro Petraglia [1,†] and Stefano Loppi [3,5,†]

[1] Department of Chemistry, Life Sciences, and Environmental Sustainability, University of Parma, Parco Area delle Scienze 11/a, 43124 Parma, Italy; luca.pagano@unipr.it (L.P.); marco.bartoli@unipr.it (M.B.); alessio.malcevschi@unipr.it (A.M.); marta.marmiroli@unipr.it (M.M.); alessandro.petraglia@unipr.it (A.P.)
[2] National Interuniveritary Consortium for Environmental (CINSA), University of Parma, Parco Area delle Scienze 95, 43124 Parma, Italy
[3] Department of Life Sciences, University of Siena, Via PA Mattioli 4, 53100 Siena, Italy; riccardo.fedeli@student.unisi.it (R.F.); stefano.loppi@unisi.it (S.L.)
[4] Department of Mathematical, Physical and Computer sciences, University of Parma, Parco Area delle Scienze 7/a, 43124 Parma, Italy; michele.sidoli@unipr.it (M.S.); giacomo.magnani@unipr.it (G.M.); daniele.pontiroli@unipr.it (D.P.); mauro.ricco@unipr.it (M.R.)
[5] BAT Center-Interuniversity Center for Studies on Bioinspired Agro-Environmental Technology, University of Naples 'Federico II', 80138 Napoli, Italy
* Correspondence: andrea.vannini@unipr.it
† These authors contributed equally to the work.

The MB molecule liquid-solid equilibrium can be modelled by Equation (S1):

$$Cs = \frac{K_L\, S_m\, C_L}{1 + K_L\, C_L} \tag{S1}$$

where Cs is the amount of MB adsorbed on the solid surface at equilibrium (mol/g), C_L is the concentration of MB in the surnatant (mol/L), S_m is the apparent sorption capacity or adsorption maximum (mol/g) and K_L is the Langmuir coefficient (g/mol). The experimental points follow the adsorption Langmuir isotherm, where the amount of absorbed MB converges towards an equilibrium value. Since every MB molecule covers 1,3 nm^2 area, the SSA can be directly obtained once the total number of MB molecules adsorbed on the sample is known.

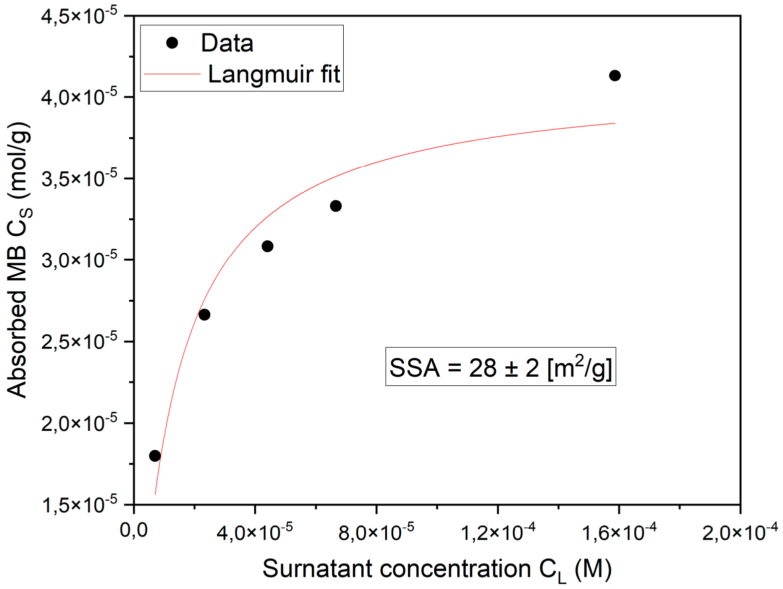

Figure S1. Langmuir isotherm fit for BC1 sample.

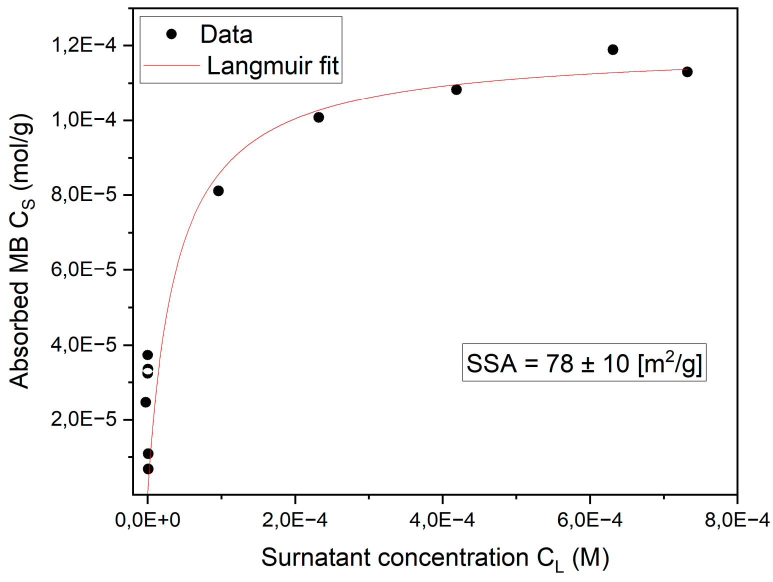

Figure S2. Langmuir isotherm fit for BC2 sample.

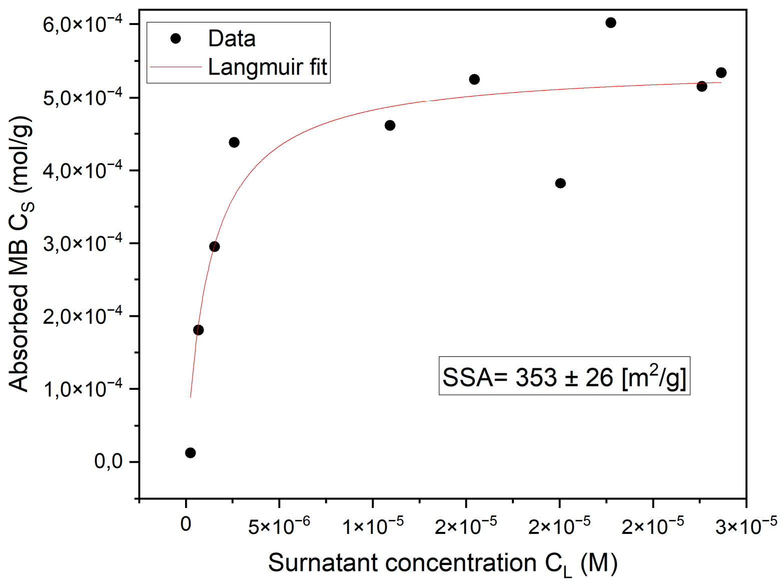

Figure S3. Langmuir isotherm fit for *Evernia prunastri* L. (Ach.) sample.
